# Cathepsin Activity-Based Probes and Inhibitor for Preclinical Atherosclerosis Imaging and Macrophage Depletion

**DOI:** 10.1371/journal.pone.0160522

**Published:** 2016-08-17

**Authors:** Ihab Abd-Elrahman, Hisanori Kosuge, Tommy Wises Sadan, Yael Ben-Nun, Karen Meir, Chen Rubinstein, Matthew Bogyo, Michael V. McConnell, Galia Blum

**Affiliations:** 1 The Institute of Drug Research, The School of Pharmacy, The Faculty of Medicine, The Hebrew University, Jerusalem, 9112001, Israel; 2 Division of Cardiovascular Medicine, Stanford University School of Medicine, Stanford, California, 94305, United States of America; 3 Department of Pathology, Hadassah Medical Center, Jerusalem, 9112001, Israel; 4 Departments of Vascular Surgery, Hadassah Medical Center, Jerusalem, 9112001, Israel; 5 Department of Pathology and Microbiology and Immunology, Stanford University School of Medicine, Stanford, California, 94305, United States of America; Ben-Gurion University of the Negev, ISRAEL

## Abstract

**Background and Purpose:**

Cardiovascular disease is the leading cause of death worldwide, mainly due to an increasing prevalence of atherosclerosis characterized by inflammatory plaques. Plaques with high levels of macrophage infiltration are considered “vulnerable” while those that do not have significant inflammation are considered stable; cathepsin protease activity is highly elevated in macrophages of vulnerable plaques and contributes to plaque instability. Establishing novel tools for non-invasive molecular imaging of macrophages in plaques could aid in preclinical studies and evaluation of therapeutics. Furthermore, compounds that reduce the macrophage content within plaques should ultimately impact care for this disease.

**Methods:**

We have applied quenched fluorescent cathepsin activity-based probes (ABPs) to a murine atherosclerosis model and evaluated their use for *in vivo* imaging using fluorescent molecular tomography (FMT), as well as *ex vivo* fluorescence imaging and fluorescent microscopy. Additionally, freshly dissected human carotid plaques were treated with our potent cathepsin inhibitor and macrophage apoptosis was evaluated by fluorescent microscopy.

**Results:**

We demonstrate that our ABPs accurately detect murine atherosclerotic plaques non-invasively, identifying cathepsin activity within plaque macrophages. In addition, our cathepsin inhibitor selectively induced cell apoptosis of 55%±10% of the macrophage within excised human atherosclerotic plaques.

**Conclusions:**

Cathepsin ABPs present a rapid diagnostic tool for macrophage detection in atherosclerotic plaque. Our inhibitor confirms cathepsin-targeting as a promising approach to treat atherosclerotic plaque inflammation.

## Introduction

Atherosclerosis is a systemic inflammatory disease with plaque formation and progression. Plaque morphology can be broadly divided into two major types, ‘stable lesions’ where the plaque is mainly fibrotic and ‘unstable lesions’ that may rupture causing acute myocardial infarction or stroke. Increased macrophage content is one of the characteristics of unstable plaques, as macrophages contribute to plaque destabilization through multiple mechanisms.

The most prominent mechanism is through degradation of the extracellular matrix resulting in a thin fibrous cap that is prone to rupture [1].

Reshaping the extracellular matrix of the plaque microenvironment is mainly controlled by matrix-metalloproteinases and cathepsin cysteine proteases that degrade collagen and elastin [2], [3]. We and others have shown that activities of both cathepsin B and S cysteine proteases are increased in macrophages from unstable human carotid plaques [4]. Targeting the highly elevated cathepsin activity may enable both detection of vulnerable plaques and focused therapy. Thus, we set out to evaluate our fluorescent cathepsin activity based probes (ABPs) as tools to detect macrophages non-invasively within atherosclerotic plaques. ABPs are small molecules that form a covalent linkage to their target enzyme in an activity-dependent manner through a reactive moiety. Quenched ABPs become fluorescent only after binding to active protease targets [5], [6]. ABPs are unique since they covalently bind their enzyme targets retaining in the active site allowing for imaging and biochemical analysis of the target enzymes [6].

It is now believed that macrophage cell depletion may be an effective approach to avoid the complications of plaque rupture [7]. We recently reported on a small molecule inhibitor of cysteine proteases that effectively deplete tumor associated macrophages [8]. Here, we compared our previously developed fluorescent cathepsin ABP, GB123, and quenched fluorescent ABP, GB137 [5] as tools for imaging cathepsin activity in mouse plaques using a non-invasive optical imaging instrument. Additionally, we investigate our cathepsin inhibitor in human atherosclerotic plaques as a potential macrophage-targeted therapy.

## Methods

### Imaging cathepsin activity in atherosclerotic mice

We used a previously described mouse carotid-ligation model [9], [10], developed for optical imaging (i.e., white coat). Eight-week-old male white FVB mice were fed high-fat diet for 4 weeks and then rendered diabetic by administration of five daily intraperitoneal injections of streptozotocin, followed by ligation of the left common carotid artery, to create macrophage-rich carotid plaques. Animals were anesthetized with inhaled 2% isoflurane for surgical procedures. Two weeks after ligation, mice were injected via tail vein using the non-quenched probe GB123 (1.2 mg/kg) or the fluorescently quenched probe GB137 (6.2 mg/kg), structures presented in S1 Fig. Mice were then imaged at 2, 4 and 8 hours post injection using FMT 2500 fluorescence molecular tomography in vivo imaging system (PerkinElmer Inc., Boston, MA) equipped with a 680 nm laser under inhalational anesthesia (2% isoflurane). Mice were sacrificed 24 hours post injection by cervical dislocation, the ligated left and non-ligated (control) right carotid artery samples were collected and imaged for *ex vivo* fluorescence, using a Maestro™ imaging system (CRI, Inc., Woburn, MA) at 649/666nm excitation/emission. Samples were incubated for 4 hours with 4% paraformaldehyde/PBS, then overnight in 30% sucrose/PBS at 4°C and embedded in OCT. Frozen samples were cut into slices using a CM 1900 cryotome (Leica Microsystems, Wetzlar, Germany). Sections, 7μm thick, were stained with primary antibodies against mouse macrophages, F4/80-PE (Invitrogen, Carlsbad, CA), and fluorescent pictures were taken with an Olympus FV10i confocal microscope (FV10i, Olympus, Tokyo, Japan). The protocol was approved by the Stanford Administrative Panel on Laboratory Animal Care (APLAC).

### Specific Macrophage Killing in Patient Samples

Carotid plaque specimens were collected from patients who underwent carotid endarterectomy at Hadassah—Hebrew University Medical Center with or without a history of cerebrovascular symptoms (i.e., amaurosis fugax, transient ischemic attack, or stroke). The study protocol was approved by the Hadassah Helsinki Review Board (approval number HMO-09-0515) with written consent as described in [4]. The carotid endarterectomy specimens were collected from 3 patients. Freshly excised tissue samples were treated with 10μM GB111-NH_2_ [6] (structure in S1 Fig) or vehicle (DMSO) for 24 hours in RPMI medium. Tissues were washed with PBS, serial frozen sections were stained with primary antibodies that were diluted in Cas-Block (Invitrogen) overnight at 4°C; monoclonal mouse anti-human CD68 clone PG-M1 (1:100, DAKO, Denmark), monoclonal rabbit anti human cleaved caspase 3 (1:400; Cell Signaling, CA, USA) and visualized with the Olympus confocal microscope. The percentage of apoptotic cells was determined by co-localization analysis using the JACoP/ImageJ program. At least two serial sections were analyzed per sample, the mean is presented ± standard Error (Statistical evaluations were done using GraphPad prism 7).

## Results

### Non-invasive imaging of plaques in atherosclerosis mouse model

We set out to analyze the capabilities of the quenched (GB137) and non-quenched (GB123) ABPs that target the activity of cathepsin B, L and S, as markers for macrophages within atherosclerotic plaques (for enzymatic data and selectivity please see [5]). Both these reagents have the same general scaffold; they are labeled with Cy5 and have a reactive acyloxymethyl ketone warhead, the primary difference is the presence of a QSY21 quenching group on the acyloxy leaving group of GB137.

**Fig 1:**
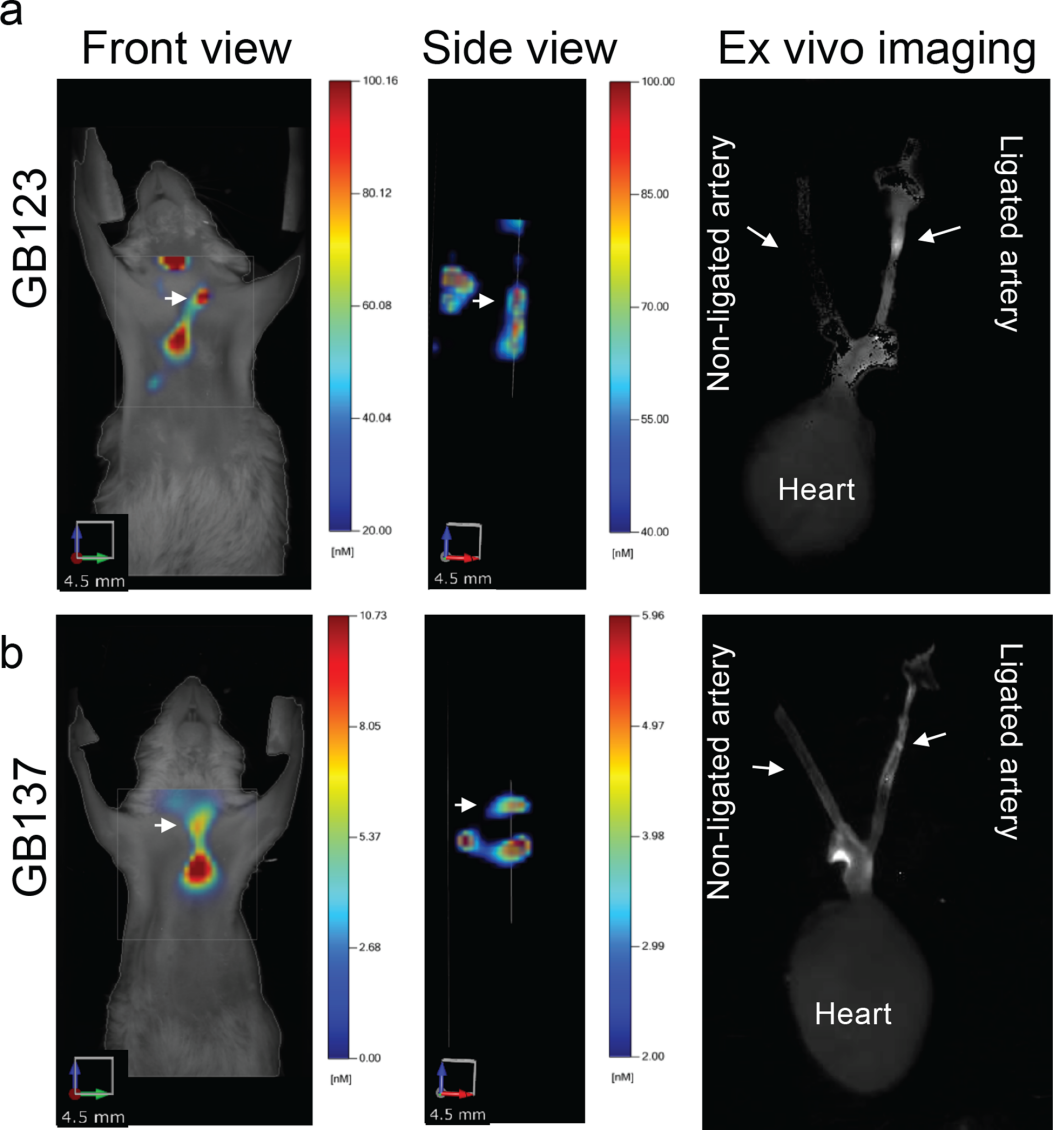
Non-invasive imaging of plaques in murine atherosclerosis. Diabetic, fat-fed mice with a ligated carotid artery were injected with non-quenched probe GB123 or quenched probe GB137 as indicated. Fluorescent molecular tomography (FMT) was used to monitor and follow the pharmacokinetics and signal accumulation in plaques. **(a, b)** Left images: front overlay of fluorescence and bright field. Middle images: side view of fluorescence alone. These images show strong fluorescence signal (arrows) (GB123 at 4 hours and GB137 at 2 hours post probe injection) around the ligated left carotid artery. Right images show *ex vivo* fluorescent image of excised heart and carotid arteries (ligated artery is marked).

*In vivo* imaging of mouse carotid arteries using FMT showed a clear signal from the non-quenched probe GB123 in the macrophage-rich ligated left carotids but not in the non-ligated (control) right carotids, at four hours post injection. We also observed signal generated by the probe in the lymph nodes as expected and in the aortic arch and heart as predicted from the disease progression (Fig 1A). Using the quenched probe GB137, we observed signal already two hours after probe injection (Fig 1B), and it was more specifically localized to the carotid lesion than GB123, demonstrating expedited and more accurate probe signal. Tomographic rotation movies of fluorescence in the chest area of mice treated with the fluorescent probes are shown in S1 Movie, 8 hours post GB123 injection and S2 Movie, 4 hours post GB137 injection.

**Fig 2:**
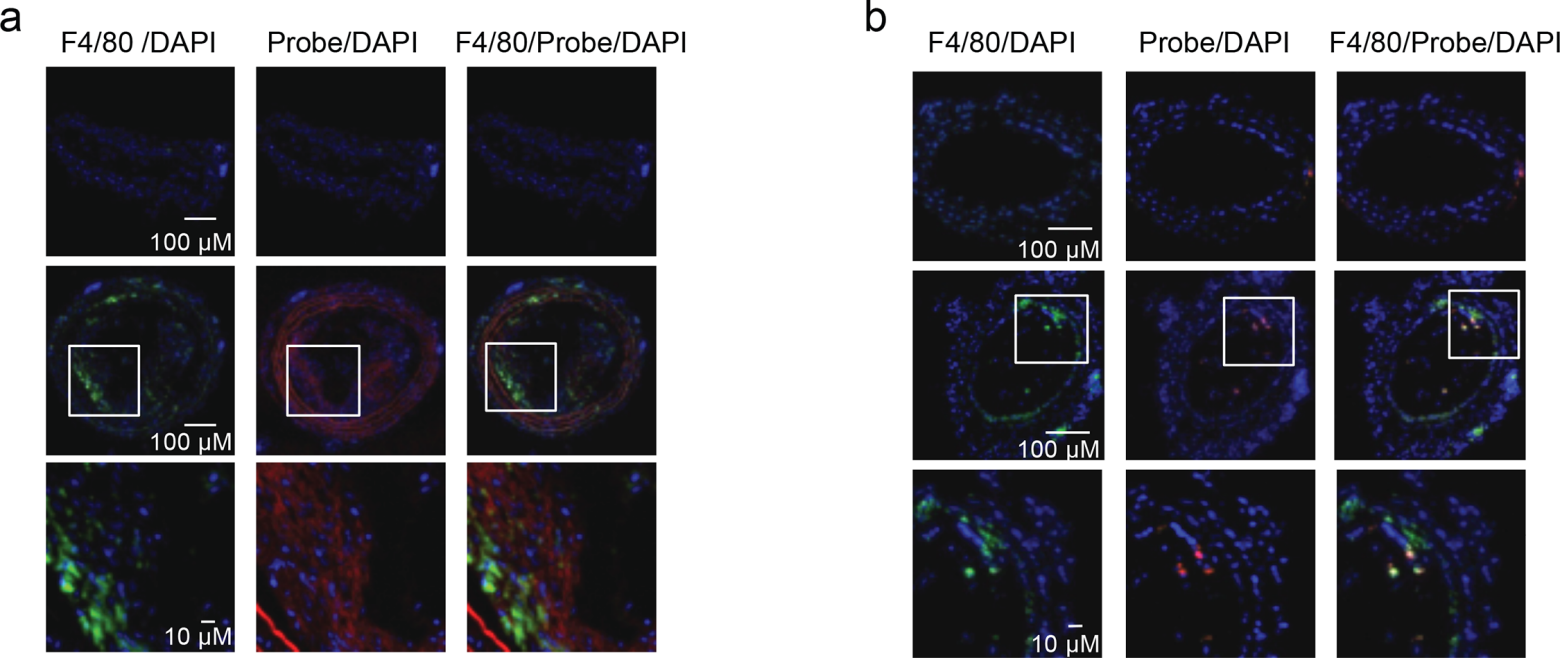
Macrophage labeling with fluorescent activity based probe. Ligated and control carotid arteries from mice treated with GB123 (**a**) or GB137 (**b**) (described in Fig 1) were embedded in OCT and serial sectioned. Samples were stained for F4/80, a macrophage marker, and scanned by a confocal microscope: DAPI (blue), Cy5 labeled by probe (red), F4/80 (green), yellow color is overlay of red and green fluorescence. Cathepsin probes were found to co-localize with F4/80 macrophages.

We confirmed the *in vivo* observation by *ex vivo* imaging of isolated hearts and carotid arteries from GB123-treated (Fig 1A right) and GB137-treated (Fig 1B right) mice. There was a clear fluorescent signal from ligated carotid arteries but a very weak signal from non-ligated carotid arteries, further demonstrating the specificity of the probe. To verify that the fluorescent signal observed in Fig 1 originated from the atherosclerotic plaque, mice were imaged after the heart and carotid arteries were removed, and no Cy5 signal was observed. Furthermore, we analyzed both the ligated and non-ligated carotid arteries using fluorescent microscopy and found that the majority of probe signal co-localized with F4/80-positive macrophages (Fig 2). Interestingly, GB123 showed greater medial fiber binding than GB137. Taken together, both our ABP (GB123) and qABP (GB137) demonstrate unique non-invasive imaging capabilities for atherosclerotic plaques, with the qABP showing superior capabilities in generating specific and rapid signaling.

### Cathepsin inhibitor induces selective macrophage apoptosis

**Fig 3:**
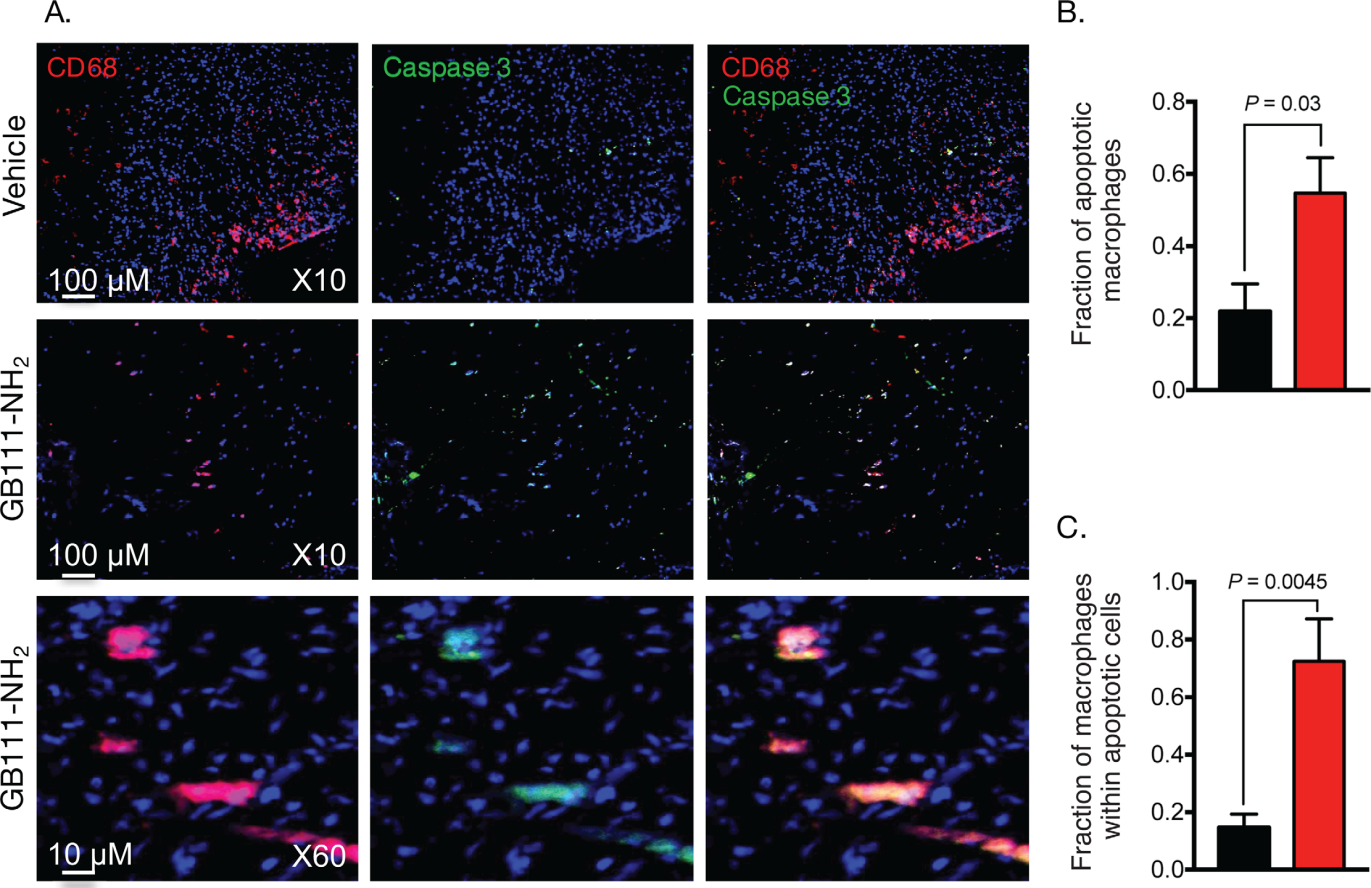
Cathepsin inhibitor induces specific macrophage apoptosis. Freshly excised human atherosclerotic tissue samples were treated with the cathepsin inhibitor GB111-NH_2_ for 24 hours. Serial frozen sections were stained for CD68 and cleaved caspase-3 and visualized by a confocal microscope: DAPI (blue), cleaved caspase-3 (green), CD68 (red), yellow color is overlay of red and green fluorescence. GB111-NH_2_ was found to induce specific macrophage cell death (a). Co-localization analysis of CD68 and cleaved Caspase 3 positive cells. Bar graphs present the fraction of apoptotic macrophages out of total CD68 population (b) and the fraction of macrophages out of total apoptotic cells is shown in (c). Data is mean ± SEM (n = 3).

We have previously shown in tumors that GB111-NH_2_, our small molecule cathepsin B, L and S inhibitor, induces M2 macrophage cell death, due to oxidative stress [8]. Since we determined that the M2 macrophages from unstable plaques display elevated cathepsins activity [4] we examined if GB111-NH_2_ can act in the same manner to promote plaque macrophage cell death. For this purpose, freshly resected human carotid plaque specimens were treated with GB111-NH_2_ for 24 hours and then tissue sections were evaluated for macrophage content and caspase-3 activation as a read-out for apoptosis. The inhibitor treatment resulted in apoptosis of 55%±10% of the plaque macrophages co-localizing with activated Caspase-3, while the basal macrophage apoptosis was only 22%±7% (Fig 3). The cell killing is macrophage selective, over 70%±15% of caspase-3 positive cells were macrophage. These results suggest that broad spectrum cathepsin inhibitors can be used for targeted macrophage depletion, which could attenuate local inflammation and increase plaque stability.

## Discussion

Here we show the application of small molecule cathepsin activity-based probes for imaging inflammation of carotid plaques in a mouse model. Both GB123 and GB137 probes were found to accumulate in the macrophage-rich carotid plaques of mice and were detectable with a non-invasive FMT imaging system. The quenched probe was more rapidly detected and was highly localized to macrophages within the inflamed plaque. Previous reports describe large polymeric substrate based cathepsin probes for molecular imaging in cancer and atherosclerosis applications [11], [12], [13], nevertheless, the fluorescent ABPs are useful since they target the intracellular pool of cathepsins and enable multiple biochemical analyses in addition to molecular imaging [6]. The covalent bond of the probes with their targets allow for fluorescent microscopy in addition to FACS and gel analysis, as previously reported [4,5,14]. Most important, the ABPs presented here can be used for rapid screening of potential therapies in preclinical setting by non-invasive molecular imaging of atherosclerotic plaques.

Since cathepsins play a key role in macrophage function, blocking their activity leads to macrophage cell death [8]. Here we show that our small molecule cathepsin inhibitor GB111-NH_2_ leads to specific macrophage apoptosis. GB111-NH_2_ was recently reported to also target the glycolytic enzymes GAPDH and α-enolase further contributing to its strong cell killing effect [15]. Thus, GB111-NH_2_ may be developed further as a potential therapy for macrophage depletion to promote plaque stability.

## Conclusion

The cathepsin molecular tools presented in this paper provide significant advancements in atherosclerosis research providing a novel diagnostic method and a potential therapeutic.

## Supporting Information

S1 Fig Cathepsin Probes and Inhibitor Structures. Structures of the inhibitor GB111-NH_2_, the non-quenched activity based probe GB123 and the quenched activity based probed GB137, both probes labeled with Cy5. Compounds published in Blum et al. 2007, Nature Chem Biol 3, 10, p.668
(TIFF)

S1 Movie A three-dimensional movie of plaque labeled with GB123. Fluorescent tomographical images were acquired by a FMT system of FVB mice treated as in Fig 1, injected with the fluorescent probe GB123 (1.2 mg/kg). Fluorescent molecular tomography (FMT) was used to monitored signal accumulation in plaques and create rotational movies.
(MOV)

S2 Movie A three-dimensional movie of plaque labeled with GB137. Fluorescent tomographical images were acquired by a FMT system of FVB mice treated as in Fig 1, injected with fluorescently quenched probe GB137 (6.2 mg/kg). Fluorescent molecular tomography (FMT) was used to monitored signal accumulation in plaques and create rotational movies.
(MOV)
