# Supplementary figures and images for: Cathepsin Activity-Based Probes and Inhibitor for Preclinical Atherosclerosis Imaging and Macrophage Depletion
Source: PLoS One. 2016 Aug 17;11(8):e0160522. doi: 10.1371/journal.pone.0160522 (PMC4988760; doi:10.1371/journal.pone.0160522)

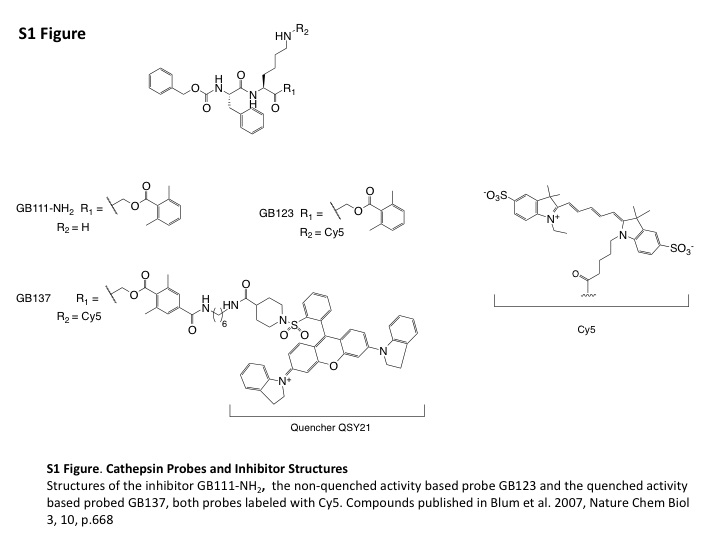

Supplement: S1 Fig — Structures of the inhibitor GB111-NH2, the non-quenched activity based probe GB123 and the quenched activity based probed GB137, both probes labeled with Cy5. Compounds published in Blum et al. 2007, Nature Chem Biol 3, 10, p.668 (TIFF) [file pone.0160522.s001.tiff]
